# Duration of Family Caregiving and Its Effects on Inflammation in the Caregiving Transitions Study

**DOI:** 10.1093/geroni/igab046.1251

**Published:** 2021-12-17

**Authors:** David Roth

**Affiliations:** Johns Hopkins University, Baltimore, Maryland, United States

## Abstract

Sustained caregiving for older adult family members with disabilities can be a chronically stressful experience that may adversely affect the health of caregivers. Systemic inflammation is thought to be one mechanism by which caregiving stress might impact health, but previous studies of inflammation in caregivers have generally found inconsistent or very small effects with questionable clinical significance when comparing caregiving and non-caregiving control samples. The Caregiving Transitions Study (CTS) enrolled 283 family caregivers and 283 carefully-matched controls from an ongoing national epidemiologic study. This population-based sample of caregivers included an unusual subsample of 32 long-term caregivers who had been providing care to the same care recipients for over 9 consecutive years. Analyses of covariance indicated that these 32 long-term caregivers had statistically significant (p < 0.05) elevations on three circulating biomarkers of inflammation – C-reactive protein, Interleukin-6, and D-dimer – compared 1) to their 32 individually-matched non-caregiving controls, and 2) to the 248 caregivers who had been providing care for less than 9 years. Covariates in the analytic models included age, sex, race, and body mass index. Similar effects were observed for caregivers of persons with or without dementia. Polynomial regression models across all caregivers revealed significant curvilinear associations of inflammation with caregiving duration. Inflammation was not markedly elevated throughout the first several years of caregiving but then begin to increase more dramatically at around 10 years of caregiving. These findings suggest that long-term caregiving, in particular, may be associated with specific physical health risks through chronically elevated systemic inflammation.

